# Oral Lesions Developed in Post‐Hematopoietic Stem Cell Transplant Patients and the Role of Cryogun Cryotherapy

**DOI:** 10.1002/cre2.70389

**Published:** 2026-06-15

**Authors:** Pei Hsuan Lu, Kelly Yi Ping Liu, Samson P. Ng, Catherine F. Poh

**Affiliations:** ^1^ Department of Dentistry National Taiwan University Hospital Yun Lin Branch Douliu Taiwan; ^2^ Department of Dentistry, National Taiwan University Hospital, College of Medicine National Taiwan University Taipei Taiwan; ^3^ School of Dentistry, Graduate Institute of Clinical Dentistry National Taiwan University Taipei Taiwan; ^4^ Faculty of Dentistry, Oral Medical and Biological Sciences University of British Columbia Vancouver British Columbia Canada; ^5^ Basic and Translation Research BC Cancer Research Centre Vancouver British Columbia Canada; ^6^ School of Biomedical Engineering, Faculty of Medicine University of British Columbia Vancouver British Columbia Canada

**Keywords:** bone marrow transplant, chronic graft‐versus‐host disease, cryotherapy, hematopoietic stem cell transplantation, oral cancer, prevention

## Abstract

**Objective:**

To investigate the development of oral premalignant and malignant lesions as a major long‐term complication following hematopoietic stem cell transplantation.

**Methods:**

This single‐center retrospective study identified 17 patients who developed 26 pathology‐confirmed oral premalignant and malignant lesions after hematopoietic stem cell transplantation, with a latency period ranged from 5 months to nearly 18 years.

**Results:**

Twelve high‐grade lesions developed in nine patients at a median of 9 years post‐hematopoietic stem cell transplantation, most commonly on the tongue. All high‐grade lesions were surgically treated, with complete response in most cases. One patient developed nodal disease 9.6 years after surgery, and another experienced local recurrence of moderate dysplasia 2 years post‐treatment, which was controlled by cryogun cryotherapy. Most patients had chronic graft‐versus‐host disease and histories of immunosuppressant therapy. Cryogun cryotherapy was used to treat 10 premalignant lesions across seven patients, involving the tongue, gingiva, and buccal or labial mucosa. This treatment achieved an 80% complete response rate with minimal scarring. Verrucous hyperplasia was the most common diagnosis (*n* = 9).

**Conclusions:**

Routine, long‐term oral screening is critical for patients after hematopoietic stem cell transplantation. Cryogun cryotherapy shows promise as an effective, minimally invasive option for managing early oral verrucous lesions.

## 1 Background

Hematopoietic stem cell transplantation (HSCT) is widely used to treat hematological malignancies as well as nonmalignant conditions such as immunodeficiencies and hemoglobinopathies (Copelan 2006). An estimated 25,000 new HSCT procedures are performed annually, with this number continue to rise (Gratwohl et al. 2010, 2013, 2015; Li and Sykes 2012; Niederwieser et al. 2022, 2016). Chronic graft‐versus‐host disease (cGVHD), a common post‐transplant complication, occurs when alloreactive donor T cells attack host tissues. Clinically and histologically, cGVHD resembles autoimmune disease such as scleroderma, primary biliary cirrhosis, Sjögren's syndrome, systemic lupus erythematosus and muco‐cutaneous lichenoid lesions (Copelan 2006; Jagasia et al. 2015; Lee et al. 2003; Tyndall and Dazzi 2008). Nearly half of transplant recipients who survive beyond 1 year develop cGVHD. cGVHD can affect nearly every organ system. The oral cavity is among the most commonly affected sites (Janowiak‐Majeranowska et al. 2022), with approximately 80% of cGVHD patients exhibit oral manifestations, including lichenoid lesions, pemphigoid‐like vesicles, and xerostomia (Eggleston et al. 1998; Dean and Sroussi 2022). Its incidence is increasing due to improved post‐transplant survival, older recipient age, and increased use of peripheral blood mobilized stem cells (Arai et al. 2015; Pidala et al. 2024).

Due to prolonged life span and long‐term immunosuppression, the development of secondary malignancies has increasingly become a major long‐term complication for HSCT survivors, with 2%–6% developing secondary tumors within 10 years, and up to 6%–13% by 15 years post‐transplant (Baker et al. 2003; Curtis et al. 1997; Mawardi et al. 2011). Squamous cell carcinomas of the skin and oral cavity account for one‐third of post‐HSCT secondary solid tumors, with oral SCC (OSCC) representing about half of those cases with ventrolateral tongue as the most frequently affected site (Montebugnoli et al. 2011; Park et al. 2026).

Recent studies emphasizes that oral cGVHD represents a chronic inflammatory microenvironment characterized by epithelial damage, immune dysregulation, and impaired mucosal repair, which may predispose to malignant transformation and the development of secondary OSCC (Curtis et al. 1997; Mawardi et al. 2011; Monteiro et al. 2024; Ichikawa et al. 2026). In a multi‐center retrospective study involving 26 post‐HSCT patients with premalignant and malignant oral lesions, 92% had clinical manifestations of oral cGVHD. Among the eight patients with premalignant lesions, all presented with oral leukoplakia, of which three had verrucous hyperplasia on the buccal mucosa, hard palate, or gingiva, and five had low‐grade dysplasia on tongue or the lower lip. In addition, two cases of verrucous carcinoma (VC) were reported among the 18 cases of malignant lesions (Mawardi et al. 2011). The median time to diagnosis of premalignant and malignant lesions was 2.5 and 8 years post‐HSCT, respectively. Although lesions such as low‐grade dysplasia and verrucous hyperplasia are often considered indolent; however, chronic inflammation and immune dysregulation in these patients may influence the biological behavior of these lesions, altering their trajectory towards malignancy (Montebugnoli et al. 2011; Ramos‐García et al. 2021; Torres‐Pereira et al. 2008). Therefore, regular surveillance and timely intervention are essential to mitigate the risk of malignant transformation.

Cryogun therapy is a technique that delivers liquid nitrogen via a handheld spray device, allowing precise and controlled freezing of targeted lesion. This rapid freeze‐thaw cycles induce cellular destruction through ice formation and tissue necrosis. Cryogun therapy serves as a highly effective, non‐invasive alternative to traditional surgical excision that is associated with minimal bleeding, low infection risk, and limited scarring. *This technique has been used for treating abnormal tissue, such as skin cancer, warts, vascular lesions, or precancerous lesions, including oral leukoplakia* (Cheng et al. 2015; Clebak et al. 2020; Yu et al. 2014). The aim of this article is to raise awareness of oral verrucous lesions as a form of oral cGVHD and to demonstrate how cryogun cryotherapy can effectively reduce disease burden.

## 2 Methods

### 2.1 Patients

At the Oral Oncology Clinic at the BC Cancer in Vancouver, Canada, a tertiary referral center, we have been enrolling patients with OSCC and premalignant lesions for a longitudinal study on disease progression and recurrence since 1998. For this retrospective analysis, we identified 17 patients who had undergone allogeneic HSCT from either allogeneic matched unrelated donors (Allo‐MUD) or allogeneic sibling donors (Allo‐Sib), and subsequently developed oral lesions, including biopsy‐confirmed OSCC, VC, conventional dysplasia, and verrucous hyperplasia. Clinical information collected included age, sex, tobacco and alcohol consumption, underlying hematological diagnosis prompting HSCT, transplant type, history of cGVHD (oral and/or systemic), treatments for systemic cGVHD, and management of oral lesions.

### 2.2 Cryotherapy

Cryotherapy was performed using a cryogun device (Brymill, Cryogenic Systems, CT, USA) delivering liquid nitrogen directly to the lesion. The application targeted the lesional area with an additional 1 mm margin of adjacent normal tissue and was sprayed approximately 10 s. Repeated the process until a uniform white frosted plaque formed lasting for 20 s for two times. Since 2016, we have incorporated cryogun cryotherapy as a topical treatment for oral precancers at our center. For lesions larger than 2 cm, the site was divided into sections and treated sequentially to minimize discomfort. In cases involving the gingiva, gingival retraction cords were used to protect adjacent teeth from thermal injury.

### 2.3 Treatment and Response of Oral Lesions

Two types of treatment were used, including surgery and cryogun cryotherapy. The outcomes include complete response (CR), defined as 0%–15% lesion remaining at the treated site; partially response (PR), defined as 15%–50% lesion remaining at the treated site; and recurrence (REC), defined as a new clinical lesion developed from the treated site at least 6 months post‐treatment. The time to outcome is defined as the time of treatment to the last clinical follow‐up time.

## 3 Results

The 17 identified patients were mainly males (76.5%), with a median age of 42 years, ranging from 14 to 67 years old at the time of HSCT (Table 1). Four (23.5%) patients were former smokers, and none of them consumed alcohol on a daily basis. All patients underwent HSCT between 1991 and 2017 for various hematological malignancies, including two non‐Hodgkin lymphoma. Seven patients received transplants from allogeneic matched unrelated donors (Allo‐MUD), while the remaining 10 received transplants from sibling donors (Allo‐sib). In addition to oral involvement, the most commonly affected systemic organs were the skin (*n* = 10) and eyes (*n* = 10). Only two patients presented with isolated oral cGVHD without systemic involvement. The severity of systemic cGVHD varied among individuals and was managed with corticosteroids (topical, oral, or intravenous) and, in some cases, monoclonal antibody therapy. Details of systemic treatment are summarized in Table 1.

**Table 1:** Patient Demographics, hematopoietic malignancy, systemic involvement, and treatment of cGVHD.

Case	Age (at transplant)	Sex	Smoking	Alcohol	Type of hematopoietic malignancy	Type of transplant (year)	cGVHD involved organs	Previous regimen for GVHD
1	15–19*	M	Never	Non‐drinker	ALL	Allo‐sib (2003)	Oral, skin, joint	Corticosteroid, Gleevec, Rituximab
2	40–45*	M	Never	Social	BAL	Allo‐sib (2004)	Oral, skin, eye, liver	Corticosteroid, Cyclosporine
3	50–55*	F	Former	Social	FL	Allo‐sib (2003)	Oral, skin	Prednisone, Cyclosporine
4	67	M	Never	Non‐drinker	PMF	Allo‐MUD (2012)	Oral, skin, eye, liver, GI	Prednisone, Cyclosporine, Mycophenolate Mofetil, Rituximab
5	51	M	Never	Non‐drinker	CML	Allo‐sib (2008)	Oral, eye, liver	Prednisone, Cyclosporine
6	57	M	Former	Non‐drinker	AML	Allo‐sib (2008)	Oral, skin, eye, lung	Prednisone, Cyclosporine
7	57	M	Never	Non‐drinker	CLL	Allo‐sib (2001)	Oral	Prednisone
8	42	M	Never	Non‐drinker	AML	Allo‐MUD (1997)	Oral, skin	None
9	41	M	Never	Non‐drinker	ALL	Allo‐sib (2009)	Oral, eye, GI	Prednisone
10	39	M	Former	Non‐drinker	PCL	Allo‐sib (1991)	Oral	None
11	62	M	Never	Non‐drinker	CLL	Allo‐sib (2015)	Oral, skin, eye, lung, joint	Prednisone, Ruxolitinib, JAK inhibitor, Methotrexate
12	40	M	Never	Social	ALL	Allo‐MUD (2011)	Oral, skin, eye, joints	Prednisone, Cyclosporine, Rituximab, Rixolitinib, Ibrutinib
13	62	M	Never	Non‐drinker	CLL	Allo‐sib (2013)	Oral, eye, liver, GI	Prednisone, Ruxolitinib
14	28	M	Never	Non‐drinker	AML	Allo‐MUD (2015)	Oral, eye	Cyclosporin, Methotrexate
15	36	M	Never	Non‐drinker	DLBCL	Allo‐MUD (2001)	Oral, eye, liver, GI, kidney	Methotrexate, Cyclosporine, Prednisone
16	14	M	Never	Non‐drinker	AML	Allo‐MUD (2017)	Oral, skin, joint	Ruxolitinib,
								Interleukin‐2
17	48	M	Former	Social	AML	Allo‐MUD (1993)	Oral, skin, GI	Prednisone

*Note:* *These three cases also had detailed disease history presented in the result section. To avoid compromising patient's anonymity, a range of age was provided.

Abbreviations: ALL, acute lymphoblastic leukemia; Allo‐MUD, allogeneic matched unrelated donors; Allo‐Sib, allogenic sibling donors; AML, acute myeloid leukemia; BAL, acute biphenotypic leukemia; cGVHD, chronic graft‐versus‐host disease; CLL, chronic lymphocytic leukemia; CML, chronic myelogenous leukemia; DLBCL; diffuse large B cell lymphoma; F, female; FL, follicular lymphoma; GI, gastrointestinal; M, male; PCL, plasma cell leukemia; PMF, primary myelofibrosis.

**Figure 1:**
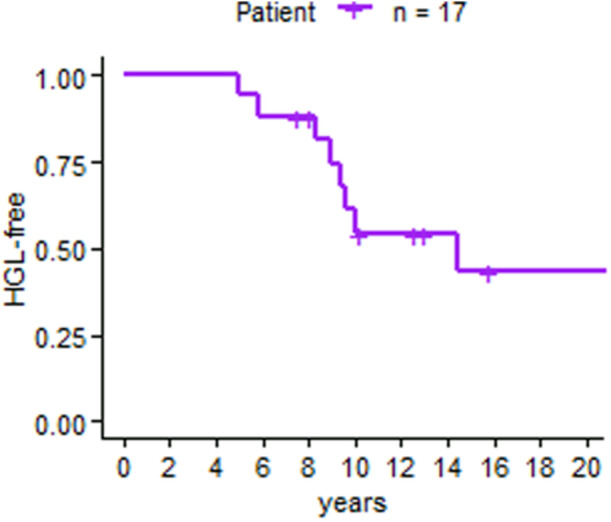
High‐grade lesion‐free survival analysis following bone marrow transplantation. Kaplan–Meier curve depicts the time to development of high‐grade oral lesions (HGLs, severe dysplasia, carcinoma in situ. or squamous cell carcinoma) among 17 transplant recipients and shows patient‐level HGL‐free survival, where an event is defined as any occurrence of any HGL in a patient with a median time of 9 years with interquartile range (ICR) of 9–10 years post‐HSCT. Censoring for last follow‐up is indicated by tick marks. The *x*‐axis represents years since transplantation, and the *y*‐axis represents the proportion of patients remaining free of HGLs. HGL: severe dysplasia, carcinoma in situ, verrucous carcinoma, or squamous cell carcinoma.

There were oral cGVHD developed at 41 anatomical sites, and the time from HSCT to the development oral cGVHD ranged from 5 months to 18 years, with a median latency of 3 years (Table 2). The most frequently involved oral sites were the tongue (*n* = 16) followed by buccal mucosa (*n* = 9), gingiva (*n* = 8), labial mucosa (*n* = 5), and hard palate (*n* = 3). Nine patients developed a total of 12 high‐grade lesions (HGLs), including 9 severe dysplasia/carcinoma in situ, 2 VCs, and one OSCC at a median time of 9 years with interquartile range of 9–10 years post‐HSCT (Figure 1). Among these HGLs, tongue was the most common site (*n* = 10), including three at dorsal tongue. All HGLs were managed surgically with good local control. One case (Case 3) developed local recurrence of verrucous hyperplasia at 8 years of follow‐up, and one case (Case 8) developed cervical nodal disease at 9.6 years without local recurrence. The patient died of disease. There was no significant difference in age at transplant, type of transplant, time from HSCT to onset of oral cGVHD, or time to diagnosis of HGLs between those developed HGLs and those with verrucous hyperplasia, low‐grade dysplasia, or lichenoid mucositis.

**Table 2:** Oral chronic graft‐versus‐host disease.

Case	Post‐HSCT oral cGVHD (years)	Oral GVHD sites	Post‐HSCT biopsy (years)	Biopsy site	Diagnosis	Treatment	Outcome (years)
1	7.0	Tongue	19.0	Dorsal tongue	—	Cryotherapy x1	CR, 2
		Buccal mucosa		Labial mucosa	VH	Cryotherapy x1	CR, 1.9
		Labial mucosa		Left buccal mucosa	LM	Cryotherapy x1	PR, 0.8
2	5.0	Gingiva	20.0	Gingiva	VH	Cryotherapy x2	CR, 3.4
		Buccal mucosa		Buccal mucosa	VH	Cryotherapy x2	CR, 1.3
3	3.0	Tongue	9.0	Lateral tongue	D3	Surgery	REC, 8.0
				Lateral tongue	D2/VH	Cryotherapy x2	CR, 10.8
		Buccal mucosa Gingiva					
		Labial mucosa		Labial mucosa	VH	—	F/U, 10.8
4	5.0	Buccal mucosa					
		Gingiva	10.0	Gingiva	VH	Cryotherapy x1	PR, 1.9
		Hard palate					
5	3.0	Tongue	9.0	Lateral tongue	VH	Cryotherapy x2	CR, 6.6
				Dorsal tongue	VC	Surgery	CR, 6.3
		Hard palate					
6	1.0	Tongue	10.0	Lateral tongue (Lt)	CIS	Surgery	CR, 12.2
				Lateral tongue (Rt)	D3	Surgery	CR, 6.0
7	6.0	Tongue	10.0	Lateral tongue (P)	CIS	Surgery	CR, 13.4
				Lateral tongue (A)	D3	Surgery	CR, 1.8
		Labial mucosa					
8	9.0	Tongue	9.0	Dorsal tongue	SCC	Surgery	CR, 9.6 (developed cervical nodal disease with no local REC)
9	2.0	Gingiva	13.0	Gingiva	VH	Cryotherapy x2	CR, 1.3
		Buccal mucosa					
10	1.0	Tongue	15.0	Dorsal tongue	VC	Surgery	CR, 6.4
11	2.0	Tongue	5.0	Lateral tongue	CIS	Surgery	CR, 4.7
		Gingiva		Gingiva	VH	Cryotherapy x1	CR, 1.4
		Hard palate					
		Labial mucosa					
12	2.0	Tongue	9.0	Lateral tongue	D2	—	FU, 3.2
		Buccal mucosa		Buccal mucosa	VH	—	FU, 6.8
13	0.5	Tongue		Buccal mucosa	VH	—	F/U, 4.0
		Buccal mucosa	7.0				
14	1.0	Tongue	4.0	Lateral tongue	LM	—	F/U, 5.8
15	15.0	Tongue	24.0	Lateral tongue	D3	Surgery	CR, 0.1
		Gingiva					
		Labial mucosa					
16	0.5	Tongue	7.0				
		Buccal mucosa					
		Gingiva		Gingiva	VH	—	F/U, 0.5 (2025/01)
17	9.0	Buccal mucosa	9.0	Buccal mucosa	CIS	Surgery	CR, 13.3
		Gingiva		Gingiva	CIS	Surgery	CR, 2.8

Abbreviations: A, anterior; cGVHD, chronic graft‐versus‐host disease; CIS, carcinoma *in situ*; CR, complete response; D2, moderate epithelial dysplasia; D3, severe dysplasia; F/U, clinical monitoring at the last follow‐up; HSCT, hematopoietic stem‐cell transplantation; LM, lichenoid mucositis; Lt, left; P, posterior; PR; partially response (any residual lesion); REC, local recurrence; Rt, right; VC, verrucous carcinoma; VH, verrucous hyperplasia.

Among 17 patients, seven patients (Cases 1, 2, 3, 4, 5, 9, and 11) received one or two sessions of topical cryogun cryotherapy across 10 lesional sites, including 4 gingival lesions, 3 tongue lesions, and 3 labial or buccal mucosa. The most common diagnosis was verrucous lesions (*n* = 9). One recurrent tongue surgical case (Case 3) was controlled with two treatments of cryotherapy with no recurrence over 10 years of follow‐up. The topical treatment achieved a CR of 80% of the lesions (Table 2). For the two cases showing PR, Case 1 was planning for the second treatment and Case 4 showed diffuse thin homogenous leukoplakia following the first treatment. Three representative cases are detailed below to illustrate treatment response.

### 3.1 Case 1

**Figure 2:**
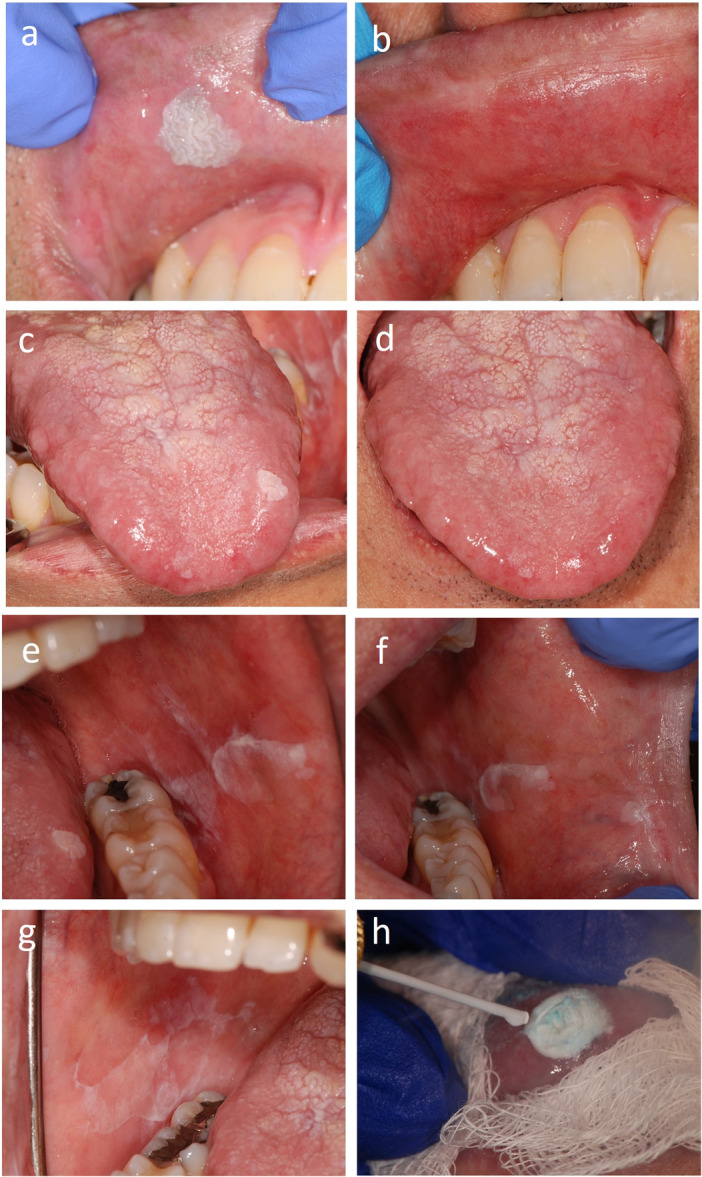
Intraoral lesions of Case 1: verrucous white lesion on the right upper labial mucosa (a), left anterior dorsal tongue (c), and bilateral buccal mucosa (e, g). Following one session of cryogun cryotherapy per site, complete response and partial response are observed on the right upper lip (b), left anterior dorsal tongue (d), and left buccal mucosa (f). A white frosted plaque is visible during cryotherapy application of the upper lip lesion (h), indicating successful freeze.

A male patient in his teens diagnosed with precursor B‐cell acute lymphoblastic leukemia (B‐ALL), underwent allogeneic peripheral blood stem cell transplantation from his sister. He later developed cGVHD affecting the skin and joints, which was managed with imatinib, corticosteroid, and rituximab. At age 41—approximately 19 years post‐transplant—he presented with multiple oral lesions on the upper right labial mucosa, dorsal tongue, and bilateral buccal mucosa, consistent with oral cGVHD (Figure 2a,c,e,f). The discrete verrucous lesion on the upper right upper labial mucosa, which the patient reported as persistent for at least 2 years, was biopsy‐confirmed verrucous hyperplasia (Figure 2a). He received one session of cryotherapy each to the upper labial mucosa, the left anterior dorsal tongue, and the right buccal mucosa. Follow‐up showed complete remission or partial resolution of among the treated lesions (Figure 2b,d,f).

### 3.2 Case 2

**Figure 3:**
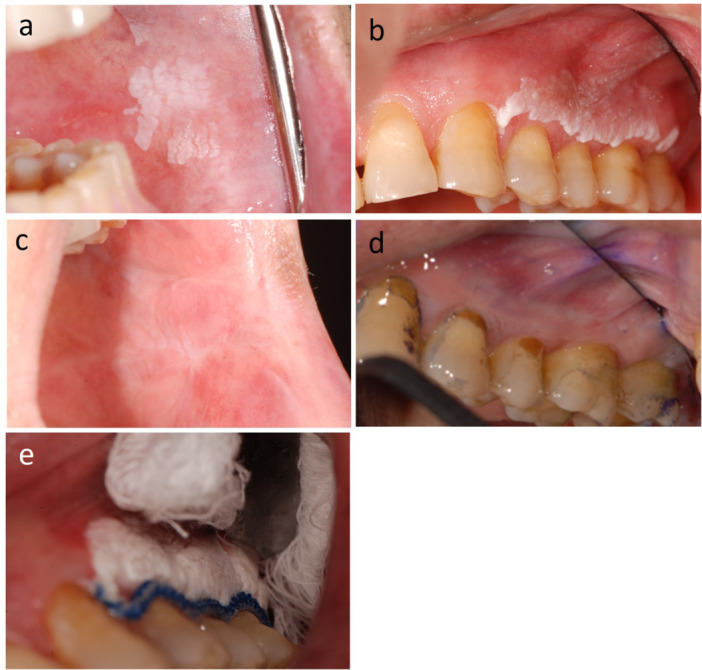
Intraoral lesions of Case 2: verrucous leukoplakia on the left buccal mucosa (a) and upper left buccal gingiva extending to buccal vestibule (b). After cryotherapy, complete response was noted at 1‐year follow‐up on both sites (c, d). A white frosted plaque appears on the gingiva after cryogun application with a blue gingival retraction cord in place to protect adjacent teeth (e).

A male patient in his 40s was, diagnosed with acute biphenotypic leukemia (BAL), underwent allogeneic bone marrow transplantation from his brother. Post‐transplant, he developed cGVHD involving the skin, eyes, liver, and oral cavity, managed with systemic corticosteroid and cyclosporine. Seven years post‐transplantation, verrucous lesions developed on the left buccal mucosa and the upper left buccal gingiva. Biopsies from both sites confirmed verrucous hyperplasia. The lesions gradually thickened over the next 3 years, prompting cryotherapy (Figure 3a,b). Treatment produced favorable results, with no recurrence at 1‐year follow‐up (Figure 3c,d).

### 3.3 Case 3

**Figure 4:**
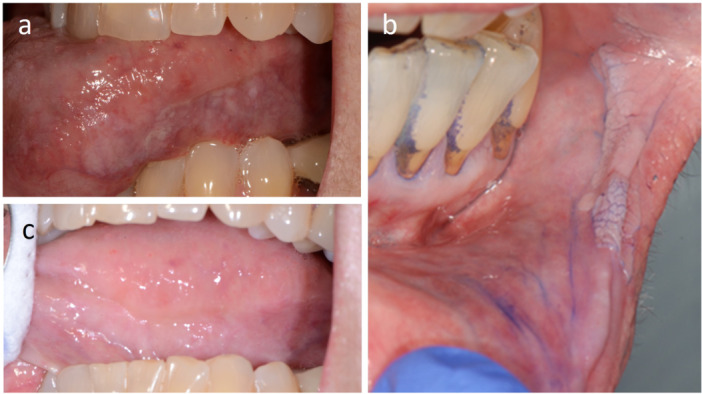
Intraoral lesions of Case 3: recurrence of a rough‐surfaced white lesion on the left ventral tongue, 2 years after excision for severe dysplasia (a), and a new verrucous lesion on the left lower lip extending to the anterior buccal mucosa (b). No recurrence was noted 7 years after cryotherapy of the tongue lesion (c).

A female patient, former smoker, in her 50s with a history of mild hypertension was diagnosed with follicular lymphoma and underwent allogeneic HSCT from a sibling donor. Twelve years post‐HSCT, she developed a lesion on the left ventral tongue, which was excised and diagnosed as severe epithelial dysplasia. Two years post‐excision, a recurrence occurred at the same site along with a new verrucous leukoplakia on the left lower lip extending to the anterior buccal mucosa (Figure 4a,b). Biopsies from both sites revealed moderate dysplasia with marked verruciform hyperorthokeratosis. The recurrent lesion on the left ventral tongue was treated with two sessions of cryotherapy, resulting in no recurrence over the following 8 years (Figure 4c).

## 4 Discussion

We reported 17 cases of oral cGVHD following HSCT. Although no definitive risk factors for the development of oral cGVHD or HGL were identified, the time to onset of oral cGVHD ranged from 5 months to 18 years, while HGLs developed between 5‐ and 25‐year post‐transplant with the 9th and 10th years at the highest risk. One patient developed a recurrent lesion at the same site, two developed two HGLs at different oral sites. These findings are consistent with a large multicenter cohort study involving 19,229 patients (Curtis et al. 1997), which showed that individuals post‐HSCT have a significantly increased risk to develop secondary malignancy—particular in the oral cavity, which carries the second highest risk, with an 11.1‐fold increase compared to the general population. Importantly, only one patient (Case 8) developed cervical lymph node metastasis, and no patients required adjuvant therapy beyond surgery. This highlights the value of long‐term oral screening, particularly in patients with a history of oral cGVHD, to enable early detection and localized treatment.

Oral cGVHD is the second most commonly affected sites after skin (Deeg and Antin 2006; Ferrara et al. 1999). Conventionally, oral cGVHD presents as lichenoid lesions that cause pain and sensitivity and may affect any oral sites. In our study, nine patients developed HGL, though only six reported mild discomfort—typically on the lateral‐ventral tongue. The remaining HGLs, as well as lower‐grade lesions, presented as painless plaques. Given that oral involvement is reported in up to 80% of patients with cGVHD, routine dental consultation should be considered when cGVHD is diagnosed in other organs to ensure early detection and management (Arai et al. 2011; Flowers et al. 2002).

Cryotherapy has long been used to manage various cutaneous and oral mucosal conditions, including premalignant lesions (Krishnan et al. 2022; Lin et al. 2012; Prasad et al. 2009; Świątkowski et al. 2024). Cryogun‐based delivery of liquid nitrogen is regarded as more precise and efficient than other methods (Lin et al. 2012). Although cryotherapy is cost‐effective and relatively simple, it is technique sensitive. Achieving a rapid and adequate freeze is critical for therapeutic success. In our experience, the key to success is to treat with a 10‐s interval until a white frosted patch forms and persists for at least 20 s (Figure 2g,e). Gingival lesions in close proximity to teeth may result in tooth sensitivity. To mitigate this, gingival retraction cords can be used during cryotherapy to provide a buffer between the lesion and the teeth (Figure 2e), or excisional biopsy can be performed to create a 1‐mm clearance.

Although lesions such as low‐grade dysplasia and verrucous hyperplasia are often considered indolent, their progression potential and risk of recurrence following malignancy underscore the need for diligent screening and management in post‐HSCT patients—particularly those with oral cGVHD. In our cohort, cryogun cryotherapy provided effective local control with minimal morbidity, particularly for well‐demarcated lesions. Its ability to be delivered in an outpatient setting, with rapid healing and low risk of scarring or infection, makes it especially suited for immunocompromised individuals who may not tolerate repeated surgical interventions. Importantly, cryotherapy may also serve as a proactive strategy during surveillance visits—allowing clinicians to manage suspicious lesions promptly and preventing potentially delay or progression to high‐grade dysplasia or carcinoma. As such, cryogun therapy complements the goals of long‐term surveillance in high‐risk post‐transplant populations.

In our experience, discrete, well‐demarcated lesions—particularly on the gingiva or dorsal tongue—respond better to cryotherapy and result in less post‐treatment discomfort. Diffuse lesions with ill‐defined borders respond less predictably. Consequently, the first‐line approach for such lichenoid mucositis should remain regular monitoring and inflammation control. Common post‐treatment effects include local pain, mild swelling, and, rarely, pyogenic granuloma formation (Gage et al. 1965). Discomfort is typically manageable with topical analgesics or over‐the‐counter pain medications. Swelling usually resolves within a few days and can be alleviated with ice packs. If pyogenic granuloma develops, it can be easily removed via excisional biopsy. Most patients fully recovered within 3 weeks, with minimal or no scar formation. Given patients' underlying systemic conditions, recurrence following cryotherapy is possible, underscoring the need for ongoing oral surveillance. The primary goal of cryotherapy in this setting is to reduce disease burden and prevent lesion progression in both clinical size and histopathology grade.

This study has several limitations that should be acknowledged. First, as a single‐center, retrospective analysis based on a specialized oral dysplasia clinic, there is an inherent selection bias towards patients referred for advanced or persistent lesions, which may not reflect the broader population of post‐HSCT patients. Second, although we included patients treated with either cryotherapy or surgery, the non‐randomized design limits the strength of comparative conclusions. Treatment decisions were based on clinical presentation, lesion location, and patient preference, which may introduce confounding factors. Third, the sample size—though notable for such a rare post‐HSCT complication—remains small, which restricts statistical power. Interestingly, several patients who developed CIS (Cases 7 and 11) and SCC (Case 8) denied tobacco and alcohol consumption. This suggests that alternative mechanisms, including chronic mucosal inflammation associated with cGVHD and prolonged immunosuppression, may play a role in carcinogenesis in these patients. Larger multicenter studies to further investigate risk factor profiles may be valuable to verify such correlations. Nevertheless, the findings highlight the prevalence of painless oral cGVHD lesions that may carry malignant potential and the clinical value of early intervention.

In summary, oral cGVHD is a relatively common and often silent complication following HSCT that can develop years after transplantation. These lesions are often painless and easily overlooked. Routine long‐term screening is essential, particularly in patients with a history of systemic cGVHD. Cryogun cryotherapy offers minimally invasive, tissue‐sparring treatment for early‐stage oral verrucous lesions and may serve as a valuable adjunct to routine oral screening in post‐HSCT patients.

## Author Contributions

P.H.L. contributed to data curation, literature review, result interpretation, and writing original draft. K.Y.P.L. was responsible for case identification, data analysis, and manuscript revision. S.P.N. participated in case identification and provided interpretation and feedback. C.F.P. designed the study, contributed to case identification, data interpretation, and manuscript writing. All authors reviewed and approved the final manuscript.

## Funding

The authors have nothing to report.

## Ethics Statement

The study was approved by the Research Ethics Board for Clinical Research of the University of British Columbia/BC Cancer Agency and conforms to the Declaration of Helsinki (REB# H11‐0216). All participants provided informed consent.

## Conflicts of Interest

The authors declare no conflicts of interest.

## Data Availability

The data that support the findings of this study are available from the corresponding author upon reasonable request. Materials described in the manuscript, including all relevant raw data, will be freely available to any researcher wishing to use them for non‐commercial purposes, without breaching participant confidentiality.
